# Cx43 Inhibition Attenuates Sepsis-Induced Intestinal Injury via Downregulating ROS Transfer and the Activation of the JNK1/Sirt1/FoxO3a Signaling Pathway

**DOI:** 10.1155/2019/7854389

**Published:** 2019-03-05

**Authors:** Zhaowei Zou, Bin Liu, Lisi Zeng, Xianzi Yang, Renli Huang, Cheng Wu, Huijuan Zhu, Yi Gao, Dongdong Yuan, Jinlong Yu

**Affiliations:** ^1^Department of General Surgery, Zhujiang Hospital, Southern Medical University, Guangzhou, China; ^2^Department of Emergency, Zhujiang Hospital, Southern Medical University, Guangzhou, China; ^3^Department of Abdominal Surgery (Section 2), Affiliated Cancer Hospital & Institute of Guangzhou Medical University, Guangzhou, China; ^4^Department of Medical Oncology, Affiliated Cancer Hospital & Institute of Guangzhou Medical University, Guangzhou, China; ^5^Department of Hepatobiliary Surgery II, Guangdong Provincial Research Center for Artificial Organ and Tissue Engineering, Guangzhou Clinical Research and Transformation Center for Artificial Liver, Institute of Regenerative Medicine, Zhujiang Hospital, Southern Medical University, Guangzhou, Guangdong Province, China; ^6^State Key Laboratory of Organ Failure Research, Southern Medical University, Guangzhou, China; ^7^Department of Anesthesiology, The Third Affiliated Hospital of Sun Yat-sen university, Guangzhou, China

## Abstract

Intestinal injury has long been considered to play a crucial role in the pathophysiology of sepsis and has even been characterized as the “motor” of it. Thus, we explored the effects of connexin43 (Cx43) on sepsis-induced intestinal injury in order to provide potential therapeutic strategies. Rat cecal ligation and puncture (CLP) models *in vivo* and cell models (IEC-6 cells) pretreated with LPS *in vitro* were used in the current study. Firstly, different methods, such as Cx43 inhibitors (18-*α*-GA and oleamide) or siRNA targeting Cx43 and N-acetyl cysteine (NAC) (a kind of ROS scavenger), were used to observe the effects of Cx43 channels mediating ROS transfer on intestinal injury. Secondly, the influence of ROS content on the activity of the JNK1/Sirt1/FoxO3a signaling pathway was explored through the application of NAC, sp600125 (a JNK1 inhibitor), and nicotinamide (a Sirt1 inhibitor). Finally, luciferase assays and ChIP were used to determine the direct regulation of FoxO3a on proapoptotic proteins, Bim and Puma. The results showed that sepsis-induced intestinal injury presented a dynamic change, coincident with the alternation of Cx43 expression. The inhibition of Cx43 attenuated CLP-induced intestinal injury *in vivo* and LPS-induced IEC-6 injury *in vitro*. The changes of Cx43 channel function regulated ROS transfer between the neighboring cells, which mediated the activation of the JNK1/Sirt1/FoxO3a signaling pathway. FoxO3a directly affected its downstream target genes, Bim and Puma, which are responsible for cell or tissue apoptosis. In summary, our results suggest that Cx43 inhibition suppresses ROS transfer and inactivates the JNK1/Sirt1/FoxO3a signaling pathway to protect against sepsis-induced intestinal injury.

## 1. Introduction

Sepsis, a life-threatening syndrome, is characterized by enhanced inflammatory response accompanied by multiple-organ dysfunction or failure and the most common cause of mortality in intensive care units [1]. The prevalence of sepsis and sepsis-related mortality remain high worldwide, which prompt us to further study its underlying mechanism and develop novel therapeutic strategies accordingly to improve the survival outcome of septic patients.

The intestine has always been considered to play a crucial role in the pathophysiology of sepsis and has even been characterized as the “motor” of the systemic inflammatory response. Sepsis causes multiple derangements in the intestinal epithelium, such as increased epithelial apoptosis, barrier dysfunction, and cytokine production [2]. According to the previous reports, preventing sepsis-induced intestinal apoptosis could improve survival 2- to 10-fold in septic peritonitis and pneumonia, although the mechanisms underlying this survival advantage remained unclear [3, 4]. Their results indicate that investigation about the mechanisms of sepsis-induced intestinal injury is instructive in clinics.

As the pathological changes in sepsis-induced intestinal injury are progressively aggravated, we focused on the function of gap junction (GJ), which is composed of Cx43 and played an important part in the process of damage amplification and deterioration [5, 6]. Connexins are a big family of transmembrane proteins, containing 21 isoforms, which express in all human organs and tissues. Six connexins compose a hemichannel, two of which in the neighboring cells dock together to form an integral GJ. Small molecules, less than 1 kDa, such as calcium, cyclic adenosine monophosphate, cyclic guanosine monophosphate, and glutathione, could be transferred directly through GJ and exert different functions. If this signal transduction leads to the death of adjacent cells, we call these signals as “death signals” and this effect is called “bystander effect,” which is the biological basis that Cx43 channel-mediated damage continuously deteriorated [7, 8]. However, the effects of Cx43 channels on sepsis-induced intestinal injury have never been reported.

Although the study about “death signals” has been going on for some time, the intrinsic quality of “death signals” is still controversial. According to the reports, we found that massive production of ROS is a remarkable feature of sepsis-induced intestinal injury and ROS, including oxygen radicals and nonradical compounds, is but one of the few signals that could be transmitted through Cx43 channels [9]. Thus, we postulated that ROS transferred by Cx43 channels would play an important role in sepsis-induced intestinal injury.

JNK1, as a family member of the MAPKs, is often involved in the increase of ROS, which could phosphorylate components of the activator protein transcription factor complex resulting in a change in cellular fate, such as Sirt1 [10]. Sirt1 is a NAD-dependent deacetylase that regulates a variety of signaling pathways through deacetylation of transcription factors. FoxO3a is the most important one among these transcription factors [11]. And FoxO3a belongs to the family of mammalian forkhead transcription factors and is also a key transcriptional regulator of Bim and Puma expression. It is also considered to be the most potent of the proapoptotic BH3-only proteins, due to their ability to bind to and neutralize all prosurvival BCL2 members [12, 13]. Thus, based on these findings, we postulated that Cx43 channels regulate ROS generation and distribution between intestinal epithelial cells, thus regulating the activity of the JNK1/Sirt1/FoxO3a signaling pathway, resulting in the expression of proapoptotic Bim and Puma and sepsis-induced intestinal injury aggravation.

## 2. Materials and Methods

### 2.1. Animals

Male Sprague-Dawley (SD) rats (200–250 g) were purchased from Guangzhou University of Chinese Medicine Animal Center (Guangzhou, China). All rats were kept in a 25-27°C environment with 12-h light/dark cycles and were fasted by only being allowed drinking before surgery. This study was performed following institutional criteria for the care and use of laboratory research animals. All the animal care and research protocols were approved by the Institutional Animal Care and Use Committee of Southern Medical University (Guangzhou, China) and performed in accordance with the National Institutes of Health guidelines for the use of experimental animals.

### 2.2. Cecal Ligation and Puncture (CLP) Surgery [14]

Rats were anesthetized with isoflurane inhalation. Importantly, the body temperature the rats should be kept at 36-38°C using a heating pad. Anesthetized rats were subjected to midline laparotomy. The cecum was carefully separated and then ligated just below the ileocecal valve and punctured twice with a 20-gauge needle. After that, the abdominal cavity was closed with two epithelium layers. Then rats were given fluid subcutaneous resuscitation. During the whole operation, damaging the blood vessels should be avoided.

### 2.3. Experimental Procedure and Drug Treatments *In Vivo*

SD rats were randomly divided into different groups according to the protocol of *in vivo* experiments (*n* = 8-10 per group). According to the corresponding experimental groups, rats were intraperitoneally pretreated with 18-*α*-glycyrrhizic acid (18-*α*-GA) (Sigma-Aldrich) at 30 mg/kg/day for 3 days before CLP surgery, with N-acetyl cysteine (NAC) (Sigma-Aldrich) at 200 mg/kg for 1 hour before CLP surgery, with sp600125 at 30 mg/kg/day for 3 days before CLP surgery, and with nicotinamide (Sigma-Aldrich) at 120 mg/kg/day for 3 days before CLP surgery. Then rats were sacrificed, and the small intestines were obtained for further examinations.

### 2.4. Intestinal Mucosa Collection [15]

At the end of the experiment, the whole small intestine was removed carefully and about 1 cm intestine was cut from 10 cm to terminal ileum. Then this part was fixed in 10% formaldehyde and embedded in paraffin for section. The remaining small intestine was washed thoroughly with 0°C normal saline and then opened longitudinally to expose the intestinal epithelium. After being dried with suction paper, the mucosal layer was harvested and stored at -70°C for further examination.

### 2.5. Assessment of Intestinal Histopathological Injury [15]

Hematoxylin and eosin (H&E) staining was used to evaluate intestinal histopathological injury. The histopathological score was estimated by an experienced pathologist who was blinded to all the groups according to the Chiu's standard.

### 2.6. Assessment of Lactate Dehydrogenase (LDH), Diamine Oxidase (DAO), and Intestinal Fatty Acid-Binding Protein (iFABP)

At the corresponding time point, 3 ml blood samples were taken from the abdominal aorta. Plasma was isolated from the samples by centrifugation at 1500g for 15 min at 4°C. Blood samples and tissues samples were used to detect the contents of LDH, DAO, and iFABP. LDH assay was carried out according to the manufacturer's instruction (Dojindo). DAO and iFABP were measured by commercial ELISA kits (Jetway Biotech Co. Ltd.) following the manufacturer's instructions.

### 2.7. Cell Lines [16] and Treatment

IEC-6 cells were obtained from the American Tissue Culture Collection and cultured in DMEM (Gibco), supplemented with 10% fetal bovine serum (Gibco), 1% nonessential amino acids (Sigma-Aldrich), and 1% glutamine (Sigma-Aldrich) at 37°C in a humidified atmosphere of 5% CO_2_. IEC-6 cells were pretreated with Cx43 channel inhibitors 18-*α*-GA (Sigma-Aldrich, 10 *μ*M for 1 hour), oleamide (Sigma-Aldrich, 25 *μ*M for 1 hour), NAC (Sigma-Aldrich, 10 mM for 1 hour), sp600125 (Sigma-Aldrich, 20 *μ*M for 24 hours), and nicotinamide (Sigma-Aldrich, 20 mM for 24 hours) before LPS (Sigma-Aldrich, 10 *μ*g/ml for 24 hours) treatment. The corresponding solvent of 18-*α*-GA and oleamide was dimethyl sulfoxide (DMSO) (Sigma-Aldrich). Supernatant was used to detect the content of LDH, DAO, and iFABP. The cell survival rate was tested using Cell Counting Kit-8 (CCK-8) (Dojindo) assay.

### 2.8. “Parachute” Dye-Coupling Assay [9]

GJ function was examined with “Parachute” dye-coupling assay *in vitro* as described. Cells were grown to confluence in 12-well plates. Donor cells from one well were incubated with a freshly made solution of 10 *μ*g/ml calcein-AM (Sigma-Aldrich) in growth medium for 30 minutes, at 37°C and pH 7.4. Calcein-AM was converted intracellularly into the GJ-permeable dye calcein. Unincorporated dye was removed by three consecutive washes with culture medium. The donor cells were then trypsinized and seeded onto the receiver cells at the ratio of 1 : 150 donor/receiver. The cells attached to the monolayer of receiver cells and formed GJs for 4 hours at 37°C and pH 7.4. The results were examined with a fluorescence microscope (EVOS FL, Life Technologies). The average number of receiver cells containing calcein per donor cell was calculated and considered as a measure of the degree of GJ.

### 2.9. Inhibition of Cx43 Expression by Small Interfering RNA (siRNA) Transfection

Specific siRNAs targeting rat Cx43 or FoxO3a (Thermo Fisher Scientific) were used to knockdown Cx43 and FoxO3a expression. Corresponding negative control siRNAs (Thermo Fisher Scientific) were also used (shown as NC in the figures). IEC-6 cells were transfected with Lipofectamine 2000 transfection reagent (Thermo Fisher Scientific) according to the manufacturer's instruction.

### 2.10. ROS Detection [6]

Cells were planted in 24-well plates to detect the content of ROS. Cells were washed twice with PBS and incubated in the presence of 10 *μ*M dihydroethidium (DHE) (Sigma-Aldrich) in serum-free DMEM for 30 minutes at 37°C. Stains of intracellular ROS were observed with a fluorescence microscope (EVOS FL, Life Technologies).

### 2.11. Western Blot Analysis

Western blotting followed the standard procedures as described. And the following antibodies were used: Cx43 (1 : 4000; Sigma-Aldrich), JNK1 (1 : 1000; Cell Signaling Technology), Sirt1 (1 : 1000, Cell Signaling Technology), FoxO3a (1 : 1000, Cell Signaling Technology), Bim (1 : 1000, Cell Signaling Technology), Puma (1 : 1000, Cell Signaling Technology), and *β*-actin (1 : 5000, Sigma-Aldrich). The images were scanned with the ImageJ scanning software, and the data were expressed as the values relative to the Sham or control value.

### 2.12. Luciferase Assays [17]

A dual-luciferase reporter kit (Promega Luciferase Assay System E1501) was used to detect luciferase activity. SiRNAs targeting FoxO3a (FoxO3a-siRNA1 or FoxO3a-siRNA2) were transiently transfected into IEC-6 cells with Lipofectamine 2000 (Invitrogen) according to the manufacturer's protocol. Then Bim and Puma luciferase reporter and internal control pRL-TK plasmids were transfected into the IEC-6 cells. The luciferase activities of the cells were measured using a dual-luciferase reporter assay kit (Promega). Reporter luciferase activity was normalized to Renilla luciferase activity.

### 2.13. ChIP Assays [17]

SiRNAs targeting FoxO3a (FoxO3a-siRNA1 or FoxO3a-siRNA2) were transiently transfected into IEC-6 cells with Lipofectamine 2000 (Invitrogen) according to the manufacturer's protocol. ChIP assays were carried out using a ChIP kit (Millipore) according to the instruction with the use of anti-FoxO3a (Cell Signaling Technology) antibody. DNA products from the immunoprecipitation were quantified by qRT-PCR relative to input. PCR were performed against the Bim primers (forward 5′-CAACACAAACCCAAGTCCT-3′ and reverse 5′-CATTTGCAAACACCCTCCTT-3′) or Puma primers (forward 5′-GACGACCTCAACGCACAGTA-3′ and reverse 5′-AGGAGTCCCATGATGAGATTGT-3′).

### 2.14. Statistical Analysis

Quantitative data are presented as mean ± SE. Statistical analysis was performed using SPSS 13.0 (SPSS Inc.) and SigmaPlot 10.0 (Systat Software Inc.). The Kolmogorov-Smirnov test was used to test the normality of the data. Multiple comparisons among different groups were analyzed using one-way ANOVA, followed by Tukey's post hoc test. *P* values less than 0.05 were considered statistically significantly different.

## 3. Results

### 3.1. CLP-Induced Intestinal Injury Presented a Dynamic Change

**Figure 1:**
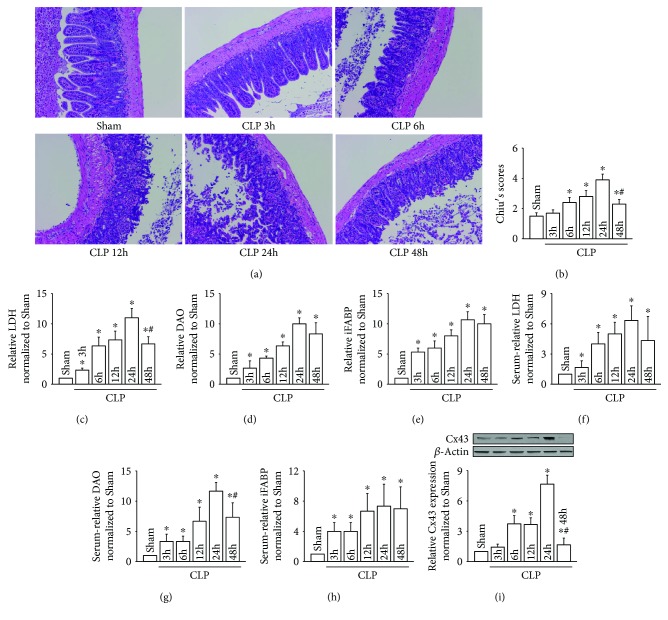
CLP-induced intestinal injuries were coincident with the changes of Cx43 expression. (a) Small intestine tissue slices were stained with H&E at different time points after CLP; (b) the histopathological score was estimated according to Chiu's standard; (c–e) levels of LDH, DAO, and iFABP in small intestine tissues; (f–h) levels of LDH, DAO, and iFABP in serum; (i) Cx43 expression of small intestine tissues at different time points after CLP. Data are shown as mean ± SD, *n* = 8-10 for each group; ^∗^*p* < 0.05 vs. the Sham group and ^#^*p* < 0.05 vs. the CLP 24 h group.

Representative images of intestinal injury caused by CLP revealing Chiu grades from 0 to 5 are shown in Figures 1(a) and 1(b), which presented a dynamic change. Twenty-four hours after CLP, intestinal injury reached the peak and then gradually recovered. This trend of dynamic change not only manifested as the intestinal pathological injury but also coordinated with the changes of LDH, DAO, and iFABP from intestinal tissues (Figures 1(c)–1(e)) or serum (Figures 1(f)–1(h)), all of which reached the peak at about 24 hours after CLP. It was consistent with the change of the intestinal pathological injury, also reflecting the degree of CLP-induced intestinal injury.

Given that Cx43 is richly expressed in the intestine and its overexpression has been shown to be related with organ damage [18], so, the expression level of Cx43 was determined after CLP. As shown in Figure 1(i), Cx43 protein was gradually increased and peaked at 24 hours after CLP, which was coincident with the most severe intestinal pathological injury and other intestinal function indicators, such as LDH, DAO, and iFABP. Results in Figure 1 provided us an evidence that Cx43 might be closely related to CLP-induced intestinal injury.

### 3.2. Cx43 Inhibition Attenuated CLP-Induced Intestinal Injury

**Figure 2:**
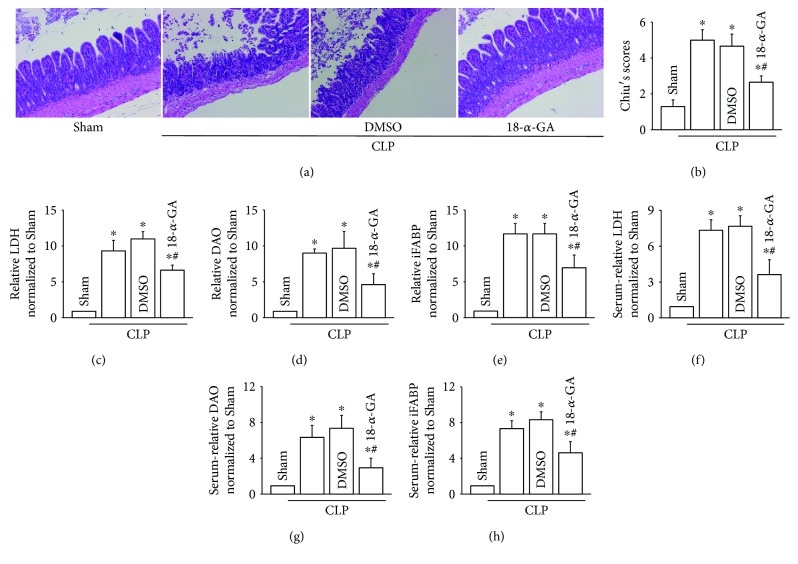
Cx43 inhibition with 18-*α*-GA attenuated CLP-induced intestinal injuries. (a) Small intestine tissue slices were stained with H&E. Rats were intraperitoneally pretreated with 18-*α*-GA (30 mg/kg/day) for 3 days before CLP surgery. (b) The histopathological score was estimated according to Chiu's standard. (c–e) Levels of LDH, DAO, and iFABP in small intestine tissues. (f–h) Levels of LDH, DAO, and iFABP in serum. Data are shown as mean ± SD, *n* = 8-10 for each group; ^∗^*p* < 0.05 vs. the Sham group and ^#^*p* < 0.05 vs. the CLP group. Vehicle control of 18-*α*-GA is DMSO.

Results in Figure 1 provided a clue that Cx43 might play an important role in CLP-induced intestinal injury. Therefore, 18-*α*-GA, considered to be a commonly used inhibitor of Cx43 channels, was employed to explore roles of Cx43 in this pathology [19]. Results in Figure 2 indicated that, after being pretreated with 18-*α*-GA, intestinal pathological injury (Figures 2(a) and 2(b)), as well as LDH, DAO, and iFABP from intestinal tissues (Figures 2(c)–2(e)) or serum (Figures 2(f)–2(h)), was attenuated obviously. The results demonstrated that Cx43 inhibition attenuated CLP-induced intestinal injury. DMSO as the vehicle control of 18-*α*-GA had no effect on the parameters.

### 3.3. Cx43 Inhibition Attenuated LPS-Induced IEC-6 Injury *In Vitro*

**Figure 3:**
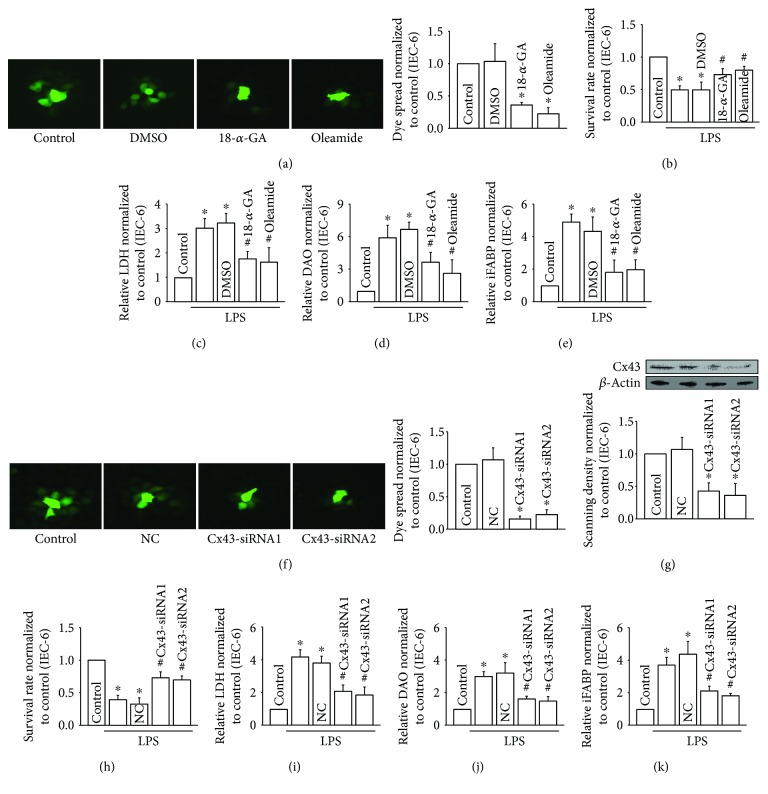
Cx43 inhibition attenuated LPS-induced IEC-6 injuries. (a) 18-*α*-GA (10 *μ*M for 1 hour) and oleamide (25 *μ*M for 1 hour) attenuated dye transfer between the neighboring IEC-6 cells with “Parachute” dye-coupling assay; (b) survival rate of IEC-6 cells pretreated with LPS (10 *μ*g/ml for 24 hours) using CCK-8 assay; (c–e) levels of LDH, DAO, and iFABP in supernatants; vehicle control of 18-*α*-GA and oleamide is DMSO; (f) Cx43 siRNAs (Cx43-siRNA1 and Cx43-siRNA2) attenuated dye transfer between the neighboring IEC-6 cells with “Parachute” dye-coupling assay; (g) Cx43 siRNAs (Cx43-siRNA1 and Cx43-siRNA2) attenuated Cx43 expression in IEC-6 cells; (h) survival rate of IEC-6 cells pretreated with LPS (10 *μ*g/ml for 24 hours) using CCK-8 assay; (i–k) levels of LDH, DAO, and iFABP in supernatants; NC: negative control; data are shown as mean ± SD, *n* = 3-5; ^∗^*p* < 0.05 vs. the control group and ^#^*p* < 0.05 vs. the LPS group.

Results in Figure 2 indicated that intestinal damage caused by CLP could be attenuated by inhibiting Cx43 function. To confirm this mechanism, we further tested it on IEC-6 cells, which is an intestinal epithelial cell line [16]. IEC-6 cells were pretreated with two commonly used inhibitors of Cx43, 18-*α*-GA, and oleamide. The results showed that the dye coupling (reflecting Cx43 channel function, detected by “Parachute” dye-coupling assay) was reduced significantly in pretreated IEC-6 cells (Figure 3(a)). At the same time, LPS-induced IEC-6 injury (Figure 3(b)), as well as LDH, DAO, and iFABP (Figures 3(c)–3(e)), was also attenuated subsequent to pretreatment with 18-*α*-GA and oleamide. In order to confirm the conclusion that Cx43 inhibition could reduce LPS-induced IEC-6 injury, two different Cx43 siRNAs (Cx43-siRNA1 and Cx43-siRNA2) were used to specifically knockdown Cx43 expression in IEC-6 cells. These two siRNAs effectively depressed Cx43 expression and dye coupling between the neighboring IEC-6 cells (Figures 3(f) and 3(g)). Certainly, as Cx43 inhibition with siRNAs, LPS-induced IEC-6 injury (Figure 3(h)), as well as LDH, DAO, and iFABP (Figures 3(i)–3(k)), was also reduced. The data indicated that Cx43 played a key role in LPS-induced IEC-6 injury.

### 3.4. Cx43 Inhibition Attenuated LPS-Induced IEC-6 Injury *In Vitro* and CLP-Induced Intestinal Injury *In Vivo* via Reducing ROS Transmission

**Figure 4:**
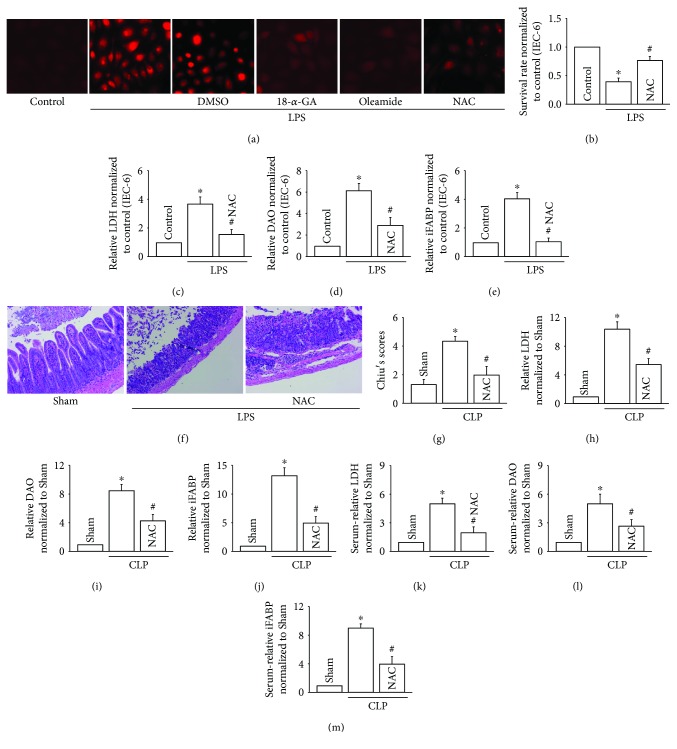
ROS inhibition improved LPS-induced IEC-6 injuries *in vitro* and CLP-induced intestinal injuries *in vivo*. (a) 18-*α*-GA (10 *μ*M for 1 hour), oleamide (25 *μ*M for 1 hour), and NAC (10 mM for 1 hour) pretreatment attenuated ROS generation and distribution between the neighboring IEC-6 cells. (b) Survival rate of IEC-6 cells pretreated with LPS (10 *μ*g/ml for 24 hours) using CCK-8 assay. (c–e) Levels of LDH, DAO, and iFABP in supernatants. In (a–e), data are shown as mean ± SD, *n* = 3 − 5; ^∗^*p* < 0.05 vs. the control group and ^#^*p* < 0.05 vs. the LPS group. (f) Small intestine tissue slices were stained with H&E. Rats were intraperitoneally pretreated with NAC (200 mg/kg) for 1 hour before CLP surgery. (g) The histopathological score was estimated according to Chiu's standard. (h–j) Levels of LDH, DAO, and iFABP in small intestine tissues. (k–m) Levels of LDH, DAO, and iFABP in serum. In (f–m), data are shown as mean ± SD, *n* = 6-8 for each group; ^∗^*p* < 0.05 vs. the Sham group and ^#^*p* < 0.05 vs. the CLP group.

As far as we know, ROS is but one of the few signals that can be transmitted through Cx43 channels, which has been reported to play an important part in multiple-organ damage [6]. Therefore, we investigated the effects of ROS mediated by Cx43 channels on LPS-induced IEC-6 injury *in vitro* and CLP-induced intestinal injury *in vivo*.  Figure 4(a) showed that both 18-*α*-GA and oleamide attenuated ROS generation and distribution effectively and NAC, a kind of ROS scavenger, also depressed the content of ROS. Of note, with the clearance of ROS, LPS-induced IEC-6 injury was attenuated, manifested as the increase of cell survival rate and the reduction of LPS, DAO, and iFABP (Figures 4(b)–4(e)). Meanwhile, in *in vivo* experiments, NAC application also attenuated CLP-induced intestinal injury, manifested as the improvement of intestinal pathological injury (Figures 4(f) and 4(g)) and the reduction of LPS, DAO, and iFABP from intestinal tissues (Figures 4(h)–4(j)) or serum (Figures 4(k)–4(m)). Thus, in this part, we concluded that ROS clearance could protect against intestinal injury *in vitro* or *in vivo* and Cx43 inhibition could attenuate ROS generation and distribution. Along with the fact that in Figures 2 and 3, we had demonstrated that Cx43 inhibition could improve intestinal injury. Therefore, we postulated that Cx43 inhibition protects against CLP-induced intestinal injury via regulating ROS generation and distribution.

### 3.5. ROS Transfer Mediated by Cx43 Channels Regulated the Activation of the JNK1/Sirt1/FoxO3a Signaling Pathway, Which Also Affected LPS-Induced IEC-6 Injury *In Vitro* and CLP-Induced Intestinal Injury *In Vivo*

**Figure 5:**
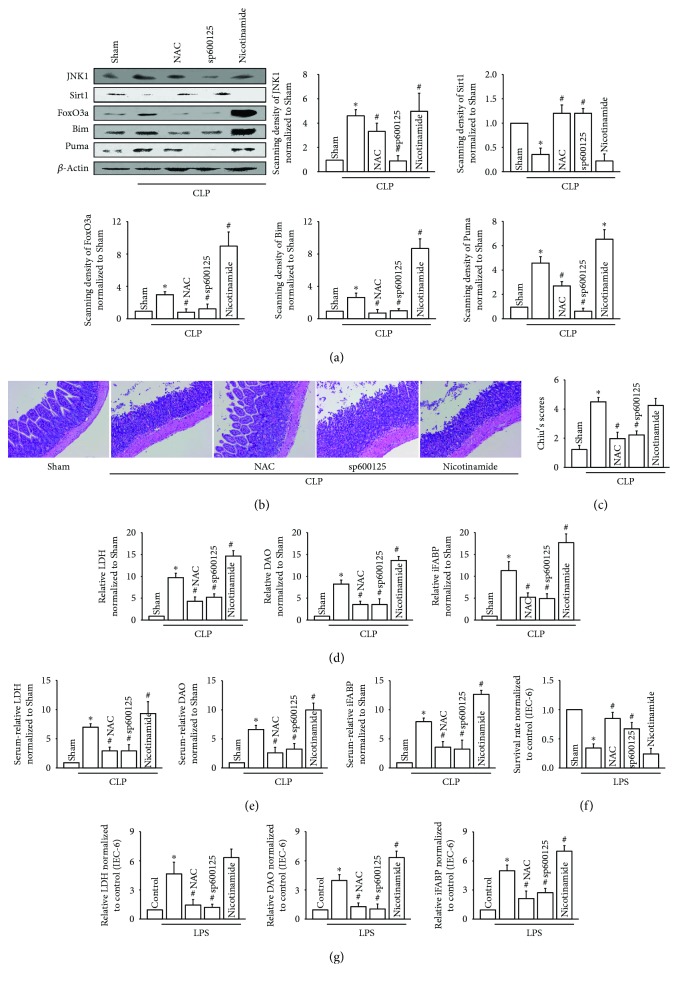
ROS regulated the activation of the JNK1/Sirt1/FoxO3a signaling pathway. (a) Effects of NAC (intraperitoneally, 200 mg/kg for 1 hour), sp600125 (intraperitoneally, 30 mg/kg for 1 hour), and nicotinamide (intraperitoneally, 120 mg/kg/day for 3 days) pretreatment before CLP surgery on JNK1, Sirt1, FoxO3a, Bim, and Puma expression. (b) Small intestine tissue slices were stained with H&E. Rats were intraperitoneally pretreated with NAC, sp600125, and nicotinamide before CLP surgery. (c) The histopathological score was estimated according to Chiu's standard. (d) Levels of LDH, DAO, and iFABP in small intestine tissues. (e) Levels of LDH, DAO, and iFABP in serum. In (a–e), data are shown as mean ± SD, *n* = 6 − 8 for each group; ^∗^*p* < 0.05 vs. the Sham group and ^#^*p* < 0.05 vs. the CLP group. (f) Survival rate of IEC-6 cells pretreated with NAC (10 mM for 1 hour), sp600125 (20 *μ*M for 24 hours), and nicotinamide (20 mM for 24 hours) before LPS (10 *μ*g/ml for 24 hours) exposure, using CCK-8 assay. (g) Levels of LDH, DAO, and iFABP in supernatants. In (f–g), data are shown as mean ± SD, *n* = 4-5; ^∗^*p* < 0.05 vs. the control group and ^#^*p* < 0.05 vs. the LPS group.

ROS could regulate many different kinds of signaling pathways, the most important one is JNK1 [20]. And in recent years, a few documents reported that the JNK signaling pathway blockade might be a rational therapeutic approach to modulate sepsis [21, 22] but the underlying mechanism was still unknown. As a protein kinase, JNK1 phosphorylates components of the activator protein transcription factor complex resulting in a change in cellular fate and this viewpoint has been widely accepted by researchers [23]. For example, it had been reported that JNK1 could phosphorylate Sirt1 and promote its enzymatic activity [24]. Thus, we investigated the effects of the JNK1/Sirt1/FoxO3a signaling pathway mediated by ROS on CLP-induced intestinal injury.  Figure 5(a) illustrated that (1) CLP led to drastic changes in JNK1/Sirt1/FoxO3a signaling pathways and its downstream genes, Bim and Puma: JNK1, FoxO3a, Bim, and Puma were all upregulated obviously, but Sirt1 was downregulated, (2) importantly, with the clearance of ROS by NAC, the expression of JNK1, FoxO3a, Bim, and Puma decreased to varying degrees and Sirt1 expression was restored to some extent, (3) the effects of JNK1 inhibition with sp600125 was similar to those of NAC caused by ROS clearance by downregulating JNK1, FoxO3a, Bim, and Puma expression, but upregulating Sirt1 expression, and (4) of note, Sirt1 inhibition with nicotinamide resulted in the dramatic increase of FoxO3a and its downstream-related genes, Bim and Puma, but had no effects on JNK1 expression, which indicated that Sirt1 was located in the downstream of JNK1. Thus, Figure 5(a) showed that the regulation of ROS on the JNK1/Sirt1/FoxO3a signaling pathway might be the intrinsic mechanism of CLP-induced intestinal injury. Therefore, we found that NAC (ROS clearance) and sp600125 (JNK1 inhibition and Sirt1 recovery) protected against CLP-induced intestinal injury, while nicotinamide (Sirt1 inhibition) caused more serious damage, manifested as intestinal pathological injury (Figures 5(b) and 5(c)) and biochemical indicator (Figure 5(d): LDH, DAO, and iFABP in tissue; Figure 5(e): LDH, DAO, and iFABP in serum). In addition, we obtained similar results in the *in vitro* experiments that LPS-induced IEC-6 injury was alleviated by NAC and sp600125 but aggravated by nicotinamide (Figures 5(f) and 5(g)).

Although the JNK1/Sirt1/FoxO3a signaling pathway was activated by sepsis, the changes of the key points were different.  Figure 5(a) showed that in CLP models, JNK1 was increased but Sirt1 was decreased. As reported, continuous activation of JNK1 resulted in the increase of ser46 phosphorylation of Sirt1, which caused proteasome-mediated Sirt1 degradation [25]. In Figure 5(a), we found that with JNK1 expression downregulated by its related inhibitor, sp600125, Sirt1 expression was restored (Figure 5(a) Sirt1 expression). Therefore, we believed that JNK1 negatively regulated Sirt1 in the model of CLP-induced intestinal injury. Sirt1 was a NAD-dependent deacetylase that could regulate the deacetylation of FoxO3a [26]. Thus, the downregulation of Sirt1 weakened the deacetylation of FoxO3a, which resulted in the activity of FoxO3a being enhanced [27]. FoxO3a was the key transcriptional regulator of Bim and Puma expression, responsible for cell or tissue apoptosis [13].

Thus, Figure 5(a) demonstrated that CLP resulted in an increase in the expression of JNK1, but a decrease in Sirt1. The downregulation of Sirt1 weakened the deacetylation of FoxO3a, which enhanced the activity of FoxO3a. When we used nicotinamide (Sirt1 inhibitor) to inhibit Sirt1 in CLP models, FoxO3a, Puma, and Bim were dramatically increased compared to those of the CLP group.

### 3.6. FoxO3a Directly Regulated Bim and Puma Expression

**Figure 6:**
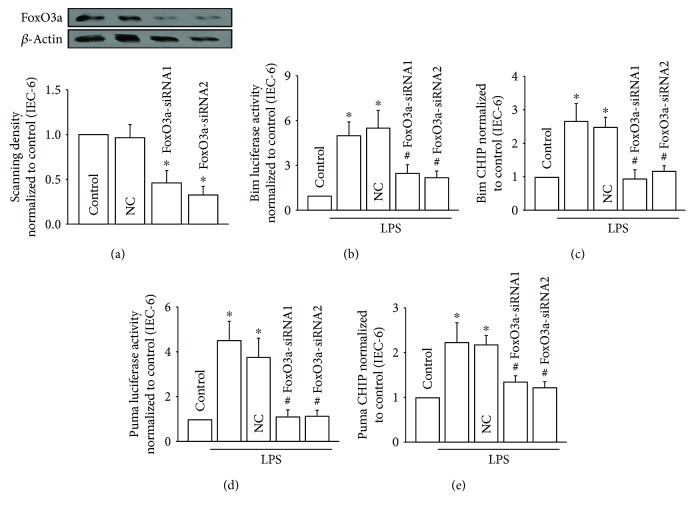
FoxO3a directly regulated Bim and Puma expression. (a) FoxO3a siRNAs (FoxO3a-siRNA1 and FoxO3a-siRNA2) attenuated FoxO3a expression in IEC-6 cells. (b) The increase of Bim luciferase activity was attenuated by FoxO3a siRNA transient transfection into IEC-6 cells with Lipofectamine 2000. (c) IEC-6 cells were pretreated with FoxO3a siRNAs (FoxO3a-siRNA1 and FoxO3a-siRNA2) and then subjected to LPS (10 *μ*g/ml) for 24 hours. ChIP analyses were performed with antibodies against FoxO3a and primers for the Bim promoter regions. (d) The increase of Puma luciferase activity was attenuated by FoxO3a siRNA transient transfection into IEC-6 cells with Lipofectamine 2000. (c) IEC-6 cells were pretreated with FoxO3a siRNAs (FoxO3a-siRNA1 and FoxO3a-siRNA2) and then subjected to LPS (10 *μ*g/ml) for 24 hours. ChIP analyses were performed with antibodies against FoxO3a and primers for the Puma promoter regions. Data are shown as mean ± SD, *n* = 3; ^∗^*p* < 0.05 vs. the control group and #*p* < 0.05 vs. the LPS group.

We have demonstrated that ROS transfer is mediated by Cx43 channels and regulated the activation of the JNK1/Sirt1/FoxO3a signaling pathway, as well as its downstream genes, Bim and Puma. In order to further confirm the regulation of FoxO3a to Bim and Puma, we designed two siRNAs targeting to FoxO3a (Figure 6(a), FoxO3a-siRNA1, FoxO3a-siRNA2) and observed the effects of FoxO3a silence on Bim and Puma. LPS exposure resulted in the increase of Bim and Puma luciferase activities, which was attenuated by FoxO3a-siRNA1 and FoxO3a-siRNA2 (Figures 6(b) and 6(d)). Next, chromatin immunoprecipitation (ChIP) assay was applied in our current study to investigate whether FoxO3a directly interacted with Bim and Puma promoters in this process. ChIP analysis revealed that the recruitment of FoxO3a protein found in the Bim and Puma promoters was both markedly reduced in response to FoxO3a-siRNA1 and FoxO3a-siRNA2 (Figures 6(c) and 6(e)). Taken together, these results supported that FoxO3a directly affects Bim and Puma expression, both were always considered to play an important part in regulating apoptosis.

## 4. Discussion

Sepsis is a life-threatening syndrome with high morbidity and mortality worldwide. Although, the intestinal injury has long been hypothesized to play a crucial role in sepsis and is frequently characterized as the “motor” of the systemic inflammatory response, the explicit mechanisms responsible for the pathogeny were not fully understood, which lead to the lack of satisfactory treatments on sepsis in clinics [2]. Thus, in the current study, we explored the underlying mechanism of intestinal injury caused by sepsis and its possible therapeutic strategies with rat CLP models *in vivo* and cell models (IEC-6 cells, a kind of intestinal epithelial cell line) pretreated with LPS *in vitro*. The current study presented that intestinal injury caused by CLP presented a dynamic change, which was coincident with the alternation of Cx43 expression. More importantly, Cx43 inhibition attenuated CLP-induced intestinal injury *in vivo* and LPS-induced IEC-6 injury *in vitro*. Our findings provide evidence that ROS transfer mediated by Cx43 channels regulated the activation of the JNK1/Sirt1/FoxO3a signaling pathway, as well as its downstream target genes, Bim and Puma, responsible for cell or tissue apoptosis which might be the underlying mechanisms.

Intestinal injury in sepsis is a progressively deteriorating the pathophysiological process. If not intervened, sepsis-induced intestinal injury, as the “motor” of the systemic inflammatory response, will lead to multiple-organ dysfunction and even organism death [28, 29]. In the present study, we found that Cx43 channels played an important part in the process of injury amplification and deterioration through “death signals” transmitting between the neighboring cells. Until now, 21 isoforms of connexins have been found; they express in all human organs and tissues with different regulations and permeabilities. Connexin isoforms form GJs which regulate intercellular signal transmission between the neighboring cells, exerting different physiological and pathological roles. These molecular signals were called “death signals,” which resulted in the cytotoxicity being amplified and deteriorated continuously. This kind of effect caused by “death signals” is called “bystander effect” [7, 30]. Compared to other connexins, Cx43 distribution is more universal and Cx43 channels have larger permeability for intercellular signals. These characteristics determine that Cx43 plays a more important role in regulating cell life activities. For example, it has been reported that Cx43 inhibition protected the brain and heart against ischemia-reperfusion injuries [31, 32]. And in our experiments, we clarified that Cx43 inhibition attenuated CLP-induced intestinal injury *in vivo* and LPS-induced IEC-6 injury *in vitro*, which prompts us that Cx43 might be a potential target to prevent further deterioration of sepsis-induced intestinal injury.

The transmission of “death signals” is always considered to be the main mechanism of Cx43 channels to exert biological effects; however, the intrinsic quality of “death signals” is still controversial. ROS, including oxygen radicals and nonradical compounds, is but one of the few signals that can be transmitted through Cx43 channels. Both oxygen radicals and nonradical compounds had much less molecular mass than the upper limit of Cx43 channel permeability [9]. Our previous study had also preliminarily confirmed the possibility of ROS as “death signals”: inhibition of ROS transmission between the neighboring cells mediated by Cx32 channels could alleviate acute kidney injury after liver transplantation [9]. And in our current study, we firstly demonstrated that inhibition of ROS transmission mediated by Cx43 channels attenuated sepsis-induced intestinal injuries.

As a strong oxidative stress signal, ROS could not only directly damage cells or tissues but also indirectly activate a series of damage-related signaling pathways [10, 20]. Combining with the fact that Cx43 channels mediated the continuous diffusion of ROS between the neighboring cells, we believed that Cx43 channel-mediated ROS transduction directly damage adjacent cells or activate downstream injury-related signaling pathways which might be one of the most important mechanisms of sepsis-induced intestinal injury continuously deteriorating. The activation of MAPKs family often involves in the increase of ROS. JNK1, a family member of the MAPKs, plays a key role in signal transduction. JNK1 is regulated by cytokines, growth factors, and cellular stresses such as heat shock, UV radiation and ROS [33]. JNK1 phosphorylated components of the activator protein transcription factor complex resulting in a change in cellular fate, such as Sirt1 [34, 35]. Sirt1 phosphorylation mediated by JNK1 had been reported to exert two opposite effects in different situations, enhancing or weakening the deacetylation of Sirt1 [25, 35], which indicated that the regulation of JNK1 on Sirt1 is not completely clear. As reported, continuous activation of JNK1 resulted in the increase of ser46 phosphorylation of Sirt1, which caused proteasome-mediated Sirt1 degradation [25]. Ultimately, the function of Sirt1 was inhibited. Results obtained from our present study were consistent with the above reports: with JNK1 expression being downregulated by its related inhibitor, sp600125, Sirt1 expression was restored. Therefore, we think that JNK1 negatively regulates Sirt1 in the model of CLP-induced intestinal injury. Sirt1 is a NAD-dependent deacetylase that regulates a variety of signaling pathways through deacetylating transcription factors such as FOXOs, p53, NF-*κ*B-mediated stress resistance, apoptosis, and inflammatory responses [26, 36]. FoxO3a belongs to the family of mammalian forkhead transcription factors. Its activity is regulated by phosphorylation and deacetylation. And Sirt1 plays a major role in the deacetylation regulation of FoxO3a. Deacetylation results in a significant decrease of FoxO3a activity. FoxO3a has also emerged as a key transcriptional regulator of Bim and Puma expression, both of which are the most potent of the proapoptotic BH3-only proteins, due to their ability to bind to and neutralize all prosurvival BCL2 members [13]. Thus, we mainly focused on the regulation of FoxO3a on Bim and Puma with different methods, such as two specific siRNAs targeting FoxO3a, luciferase assays and ChIP assays. The results indicated that FoxO3a directly affects Bim and Puma expression. Bim-mediated apoptosis and Puma-mediated apoptosis always play an important role in many intestinal diseases, such as intestinal ischemia-reperfusion injury or inflammatory bowel disease [37, 38]. It has been found that most of the intestinal epithelial exfoliated cells are apoptotic cells. Apoptosis of intestinal mucosal cells leads to damage of intestinal mucosal barrier and aggravation of intestinal injury [39, 40]. For IBD, the abnormal regeneration and apoptosis of intestinal epithelium lead to changes in the morphology of the crypt and villi and then cause changes in intestinal barrier function, which ultimately promotes the occurrence of IBD [41, 42]. In our current study, we found that with the increase of Bim and Puma expression, CLP-induced intestinal injury *in vivo* and LPS-induced IEC-6 injury *in vitro* were both deteriorated. In contrast, with the downregulation of Bim and Puma expression, intestinal injury was improved. Therefore, regulating the expression of Bim and Puma might have an important clinical significance in improving intestinal injury in sepsis.

## 5. Conclusions

We have conducted a series of *in vitro* and *in vivo* studies and firstly described a completely different new mechanism of sepsis-induced intestinal injuries. The enhancement of Cx43 channel function results in ROS increase, and redistribution regulates the activation of the JNK1/Sirt1/FoxO3a signaling pathway, as well as its downstream target genes, Bim and Puma, responsible for cell or tissue apoptosis. Because of the existence of Cx43 channels, ROS transmits continuously between adjacent cells and activated downstream of the JNK1/Sirt1/FoxO3a signaling pathway, which results in the intestinal injury continuously enlarging and deteriorating. This is more in line with the pathological process of sepsis-induced intestinal injury but also different from the traditional research about its mechanism, so we believed that our research is more instructive in clinics.

## Data Availability

All data generated or analyzed during this study are included in this published article.

## References

[1] Liu S., Xie J., Zhao B. (2018). ADAR1 prevents small intestinal injury from inflammation in a murine model of sepsis. *Cytokine*.

[2] Dominguez J. A., Samocha A. J., Liang Z., Burd E. M., Farris A. B., Coopersmith C. M. (2013). Inhibition of IKK*β* in enterocytes exacerbates sepsis-induced intestinal injury and worsens mortality. *Critical Care Medicine*.

[3] Coopersmith C. M., Stromberg P. E., Dunne W. M. (2002). Inhibition of intestinal epithelial apoptosis and survival in a murine model of pneumonia-induced sepsis. *JAMA*.

[4] Coopersmith C. M., Chang K. C., Swanson P. E. (2002). Overexpression of Bcl-2 in the intestinal epithelium improves survival in septic mice. *Critical Care Medicine*.

[5] Yuan D., Su G., Liu Y. (2016). Propofol attenuated liver transplantation-induced acute lung injury via connexin43 gap junction inhibition. *Journal of Translational Medicine*.

[6] Gu Y., Huang F., Wang Y. (2018). Connexin32 plays a crucial role in ROS-mediated endoplasmic reticulum stress apoptosis signaling pathway in ischemia reperfusion-induced acute kidney injury. *Journal of Translational Medicine*.

[7] Soares E. S., Mendonça M. C. P., Rocha T., Kalapothakis E., da Cruz-Höfling M. A. (2016). Are synchronized changes in connexin-43 and caveolin-3 a bystander effect in a *Phoneutria nigriventer* venom model of blood-brain barrier breakdown?. *Journal of Molecular Neuroscience*.

[8] Cottin S., Ghani K., Caruso M. (2008). Bystander effect in glioblastoma cells with a predominant cytoplasmic localization of connexin43. *Cancer Gene Therapy*.

[9] Luo C., Yuan D., Li X. (2015). Propofol attenuated acute kidney injury after orthotopic liver transplantation via inhibiting gap junction composed of connexin 32. *Anesthesiology*.

[10] Zhang Z., Guo M., Zhao S., Shao J., Zheng S. (2016). ROS-JNK1/2-dependent activation of autophagy is required for the induction of anti-inflammatory effect of dihydroartemisinin in liver fibrosis. *Free Radical Biology & Medicine*.

[11] Liu Z., Zhang M., Zhou T., Shen Q., Qin X. (2018). Exendin-4 promotes the vascular smooth muscle cell re-differentiation through AMPK/SIRT1/FOXO3a signaling pathways. *Atherosclerosis*.

[12] Huo L., Bai X., Wang Y., Wang M. (2017). Betulinic acid derivative B10 inhibits glioma cell proliferation through suppression of SIRT1, acetylation of FOXO3a and upregulation of Bim/PUMA. *Biomedicine & Pharmacotherapy*.

[13] Ghosh A. P., Klocke B. J., Ballestas M. E., Roth K. A. (2012). CHOP potentially co-operates with FOXO3a in neuronal cells to regulate PUMA and BIM expression in response to ER stress. *PLoS One*.

[14] Qiu R., Yao W., Ji H. (2018). Dexmedetomidine restores septic renal function via promoting inflammation resolution in a rat sepsis model. *Life Sciences*.

[15] Gan X., Su G., Zhao W., Huang P., Luo G., Hei Z. (2013). The mechanism of sevoflurane preconditioning-induced protections against small intestinal ischemia reperfusion injury is independent of mast cell in rats. *Mediators of Inflammation*.

[16] Otake K., Sato N., Kitaguchi A. (2018). The effect of lactoferrin and pepsin-treated lactoferrin on IEC-6 cell damage induced by clostridium difficile toxin B. *Shock*.

[17] Ge M., Yao W., Yuan D. (2017). Brg1-mediated Nrf2/HO-1 pathway activation alleviates hepatic ischemia-reperfusion injury. *Cell Death & Disease*.

[18] Ey B., Eyking A., Gerken G., Podolsky D. K., Cario E. (2009). TLR2 mediates gap junctional intercellular communication through connexin-43 in intestinal epithelial barrier injury. *The Journal of Biological Chemistry*.

[19] Zhang Z., Chen Y., Zhang T. (2016). Role of myoendothelial gap junctions in the regulation of human coronary artery smooth muscle cell differentiation by laminar shear stress. *Cellular Physiology and Biochemistry*.

[20] Yu J., Gao H., Wu C., Xu Q. M., Lu J. J., Chen X. (2018). Diethyl blechnic, a novel natural product isolated from *Salvia miltiorrhiza* bunge, inhibits doxorubicin-induced apoptosis by inhibiting ROS and activating JNK1/2. *International Journal of Molecular Sciences*.

[21] Bao R., Hou J., Li Y. (2016). Adenosine promotes Foxp3 expression in Treg cells in sepsis model by activating JNK/AP-1 pathway. *American Journal of Translational Research*.

[22] Pizzino G., Bitto A., Pallio G. (2015). Blockade of the JNK signalling as a rational therapeutic approach to modulate the early and late steps of the inflammatory cascade in polymicrobial sepsis. *Mediators of Inflammation*.

[23] Song Y., Liu X., Yue H., Ji J., Dou H., Hou Y. (2015). Anti-inflammatory effects of benzenediamine derivate FC-98 on sepsis injury in mice via suppression of JNK, NF-*κ*B and IRF3 signaling pathways. *Molecular Immunology*.

[24] Hwang J. S., Ham S. A., Yoo T. (2016). Upregulation of MKP-7 in response to rosiglitazone treatment ameliorates lipopolysaccharide-induced destabilization of SIRT1 by inactivating JNK. *Pharmacological Research*.

[25] Gao Z., Zhang J., Kheterpal I., Kennedy N., Davis R. J., Ye J. (2011). Sirtuin 1 (SIRT1) protein degradation in response to persistent c-Jun N-terminal kinase 1 (JNK1) activation contributes to hepatic steatosis in obesity. *The Journal of Biological Chemistry*.

[26] Ferguson D., Shao N., Heller E. (2015). SIRT1-FOXO3a regulate cocaine actions in the nucleus accumbens. *The Journal of Neuroscience*.

[27] Sun W., Qiao W., Zhou B. (2018). Overexpression of Sirt1 in mesenchymal stem cells protects against bone loss in mice by FOXO3a deacetylation and oxidative stress inhibition. *Metabolism*.

[28] Krezalek M. A., DeFazio J., Zaborina O., Zaborin A., Alverdy J. C. (2016). The shift of an intestinal “microbiome” to a “pathobiome” governs the course and outcome of sepsis following surgical injury. *Shock*.

[29] Fujii H., Takahashi T., Nakahira K. (2003). Protective role of heme oxygenase-1 in the intestinal tissue injury in an experimental model of sepsis. *Critical Care Medicine*.

[30] Kranz K., Paquet-Durand F., Weiler R., Janssen-Bienhold U., Dedek K. (2013). Testing for a gap junction-mediated bystander effect in retinitis pigmentosa: secondary cone death is not altered by deletion of connexin36 from cones. *PLoS One*.

[31] Theodoric N., Bechberger J. F., Naus C. C., Sin W. C. (2012). Role of gap junction protein connexin43 in astrogliosis induced by brain injury. *PLoS One*.

[32] Vetterlein F., Muhlfeld C., Cetegen C., Volkmann R., Schrader C., Hellige G. (2006). Redistribution of connexin43 in regional acute ischemic myocardium: influence of ischemic preconditioning. *American Journal of Physiology. Heart and Circulatory Physiology*.

[33] Syed I., Kyathanahalli C. N., Jayaram B. (2011). Increased phagocyte-like NADPH oxidase and ROS generation in type 2 diabetic ZDF rat and human islets: role of Rac1–JNK1/2 signaling pathway in mitochondrial dysregulation in the diabetic islet. *Diabetes*.

[34] Vinciguerra M., Santini M. P., Martinez C. (2012). mIGF-1/JNK1/SirT1 signaling confers protection against oxidative stress in the heart. *Aging Cell*.

[35] Nasrin N., Kaushik V. K., Fortier E. (2009). JNK1 phosphorylates SIRT1 and promotes its enzymatic activity. *PLoS One*.

[36] Cao C., Lu S., Kivlin R. (2009). SIRT1 confers protection against UVB- and H2O2-induced cell death via modulation of p53 and JNK in cultured skin keratinocytes. *Journal of Cellular and Molecular Medicine*.

[37] Wu B., Ootani A., Iwakiri R. (2004). Ischemic preconditioning attenuates ischemia-reperfusion-induced mucosal apoptosis by inhibiting the mitochondria-dependent pathway in rat small intestine. *American Journal of Physiology Gastrointestinal and Liver Physiology*.

[38] Lyu W., Jia H., Deng C., Saito K., Yamada S., Kato H. (2017). Zeolite-containing mixture supplementation ameliorated dextran sodium sulfate-induced colitis in mice by suppressing the inflammatory bowel disease pathway and improving apoptosis in colon mucosa. *Nutrients*.

[39] Grootjans J., Hodin C. M., de Haan J.–. J. (2011). Level of activation of the unfolded protein response correlates with Paneth cell apoptosis in human small intestine exposed to ischemia/reperfusion. *Gastroenterology*.

[40] Ikeda H., Suzuki Y., Suzuki M. (1998). Apoptosis is a major mode of cell death caused by ischaemia and ischaemia/reperfusion injury to the rat intestinal epithelium. *Gut*.

[41] Tang R., Yang G., Zhang S., Wu C., Chen M. (2014). Opposite effects of interferon regulatory factor 1 and osteopontin on the apoptosis of epithelial cells induced by TNF-*α* in inflammatory bowel disease. *Inflammatory Bowel Diseases*.

[42] Pedersen J., LaCasse E. C., Seidelin J. B., Coskun M., Nielsen O. H. (2014). Inhibitors of apoptosis (IAPs) regulate intestinal immunity and inflammatory bowel disease (IBD) inflammation. *Trends in Molecular Medicine*.

